# 2897. Development of a Machine Learning Modelling Tool for Predicting Incident HIV Using Public Health Data from a County in the Southern United States

**DOI:** 10.1093/ofid/ofad500.168

**Published:** 2023-11-27

**Authors:** Carlos s Saldana, Elizabeth Burkhardt, Alfred Pennisi, Kirsten Oliver, John Olmstead, David P Holland, Jenna Gettings, Pascale Wortley, Karla V Saldana Ochoa

**Affiliations:** Emory University School of Medicine, Atlanta, GA; Georgia Department of Public Health, Atlanta, Georgia; Georgia Department of Public Health, Atlanta, Georgia; Georgia Department of Public Health, Atlanta, Georgia; Georgia Department of Public Health, Atlanta, Georgia; Emory University School of Medicine, Atlanta, GA; Centers for Disease Control and Prevention, Atlanta, Georgia; Georgia Department of Public Health, Atlanta, Georgia; University of Florida, Gainesville, Florida

## Abstract

**Background:**

Machine Learning (ML) algorithms have predicted incident HIV using electronic medical record (EMR) data. We developed an ML model using de-identified public health data from a high-incidence area to predict incident HIV which could inform public health interventions such as HIV testing, education, and scale-up prevention strategies.

**Methods:**

We used de-identified public health data from Georgia’s State Electronic Notifiable Disease Surveillance System (SendSS) and Enhanced HIV/AIDS Reporting System (eHARs) from 01/2010 to 12/2021 in Fulton County - GA. Included variables are displayed in **Table 1**. We included males, 13 years of age and older. Patient's HIV status and HIV incidence during the study period were confirmed by matching individuals between the datasets. We excluded individuals diagnosed with HIV before 2010, those with an HIV diagnosis as their first sexually transmitted infection (STI) dataset entry, and those individuals with more than 10% of variables missing. We matched a social vulnerability index (SVI) to an individual census tract. We trained various ML classification models with an equal number of HIV-positive and randomly selected HIV-negative observations to balance both the training (85%) and test sets (15%) to predict incident HIV.

**Table 1.**
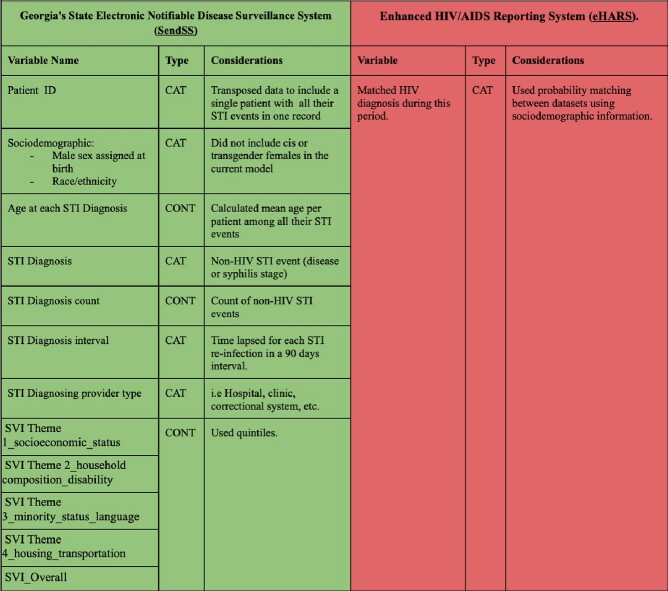
Data sources and variables included in the model From datasets in Fulton County - Georgia 2010-2021. ID=Identification SVI=Social vulnerability index STI= Sexually transmitted infection HIV=human immunodeficiency virus CAT=categorical CONT=continuos

**Results:**

Of 85,224 individuals, a total of 1,698 male individuals (2%) were confirmed positive for HIV during the study period and met our inclusion criteria. The training set included 2,896 observations (1,448 HIV+ and 1,448 HIV-) and the test set included 500 observations (250 HIV+ and 250 HIV-). Among the ML models used, Gradient Boosted Trees and Random Forest achieved an accuracy as high as 80% for correctly predicting incident HIV in the test set. The most predictive features were mean age at STI diagnosis, STI diagnosing provider type, STI diagnosis interval, SVI Theme 1, and STI diagnosis. Model performance and evaluation are presented in **Figure 1.**

**Figure 1.**
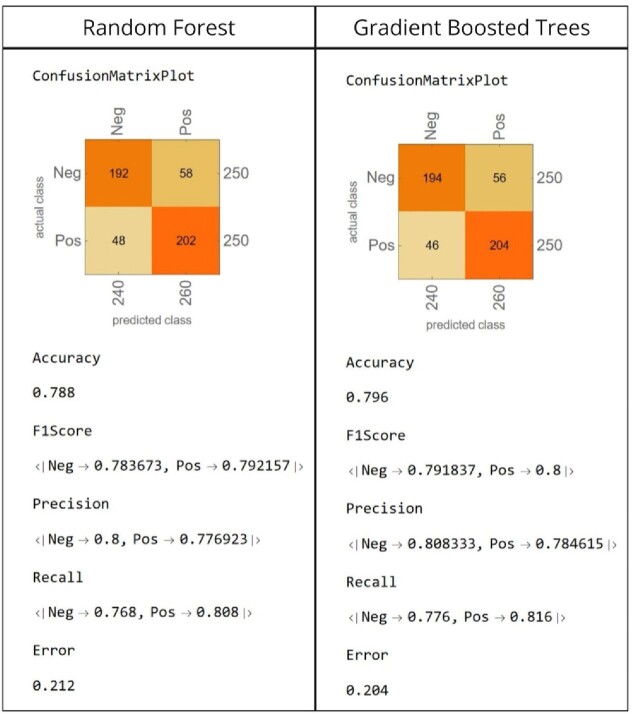
Machine Learning Classifier Performance Evaluation on Test Set.

Random Forest (RF) and Gradient Boosted Trees (GBT) confusion matrix on a test set of 500 observations. Overall both RF and GBT models achieved an overall high accuracy in correctly predicting incident HIV in 202/250 (79%) and 204/250 (80%) individuals respectively. Precision= number of true positives divided by the total number of positive predictions; Recall= Percentage of observation the model correctly identifies as belonging to their class; F-1 Score= Combined score of precision and recall.

**Conclusion:**

Our ML models can accurately predict incident HIV and can be used to customize outreach activities. The approach used is unique in that it strictly relies on de-identified STI reporting public health data, which makes it suitable for a broader population than EMR data. However, more research is needed to implement and evaluate these models in actual public health interventions.

**Disclosures:**

**All Authors**: No reported disclosures

